# EGFR marks a subpopulation of dermal mesenchymal cells highly expressing IGF1 which enhances hair follicle regeneration

**DOI:** 10.1111/jcmm.17766

**Published:** 2023-05-10

**Authors:** Min Chen, Zaoxu Xu, Yu Chen, Qingyang Yang, Ruiqing Lu, Yankai Dong, Xiaosong Li, Jundong Xie, Ren‐He Xu, Haidong Jia, Yan Kang, Yaojiong Wu

**Affiliations:** ^1^ Tsinghua‐Berkeley Shenzhen Institute Tsinghua University Shenzhen China; ^2^ State Key Laboratory of Chemical Oncogenomics, and the Institute of Biopharmaceutical and Health Engineering (iBHE), Shenzhen International Graduate School Tsinghua University Shenzhen China; ^3^ College of Life Sciences University of Chinese Academy of Sciences Beijing China; ^4^ Faculty of Health Sciences University of Macau Taipa China; ^5^ Shanghai Jahwa United Co., Ltd Shanghai China

**Keywords:** cell subpopulation, EGFR, hair follicle regeneration, *Igf1*, scRNA‐seq

## Abstract

The skin harbours transcriptionally and functionally heterogeneous mesenchymal cells that participate in various physiological activities by secreting biochemical cues. In this study, we aimed to identify a new subpopulation of dermal mesenchymal cells that enhance hair follicle regeneration through a paracrine mechanism. Integrated single‐cell RNA sequencing (scRNA‐seq) data analysis revealed epidermal growth factor receptor (EGFR) as a marker of distinct fibroblast subpopulation in the neonatal murine dermis. Immunofluorescence staining and fluorescence‐activated cell sorting (FACS) were used to validate the existence of the cell population in *Krt14‐rtTA‐H2BGFP* mouse. The difference of gene expression between separated cell subpopulation was examined by real‐time PCR. Potential effect of the designated factor on hair follicle regeneration was observed after the application on excisional wounds in *Krt14‐rtTA‐H2BGFP* mouse. Immunofluorescence staining demonstrated the existence of dermal EGFR^+^ cells in neonatal and adult mouse dermis. The EGFR^+^ mesenchymal population, sorted by FACS, displayed a higher expression level of *Igf1* (insulin‐like growth factor 1). Co‐localisation of IGF1 with EGFR in the mouse dermis and upregulated numbers of hair follicles in healed wounds following the application of exogenous IGF1 illustrated the contribution of EGFR^+^ cells in promoting wound‐induced hair follicle neogenesis. Our results indicate that EGFR identifies a subpopulation of dermal fibroblasts that contribute to IGF1 promotion of hair follicle neogenesis. It broadens the understanding of heterogeneity and the mesenchymal cell function in skin and may facilitate the potential translational application of these cells.

## 1 INTRODUCTION

Mesenchymal cells are the most abundant cell types in the skin dermis, and actively participate in skin homeostasis and repair. However, the heterogeneity and functional diversity of mesenchymal cells complicate our insights into their physiological role in skin. Over the past decade, a number of studies have uncovered several dermal mesenchymal cell lineages that have distinct functions in skin homeostasis and diseases.^1^, ^2^, ^3^ Cell lineage tracing analysis has revealed that CD26 and DLK1 are markers for papillary fibroblasts in the upper dermis and reticular fibroblasts in the lower dermis, respectively, each of which displays distinct activities in skin repair and regeneration.^1^, ^2^, ^3^ Fibroblasts derived from the lower dermis move upward during wound healing and exhibit profibrotic activities.^3^, ^4^ Indeed, subsets of dermal fibroblasts with profibrotic activities have been identified, and shown to have distinct features, including ADAM12^+^PDGFRα^+^ cells^5^ and engrailed‐1^+^ mesenchymal cells.^6^ On the other hand, studies have implied that certain dermal fibroblast subsets enhance skin regeneration, such as *Hic1* ^+^ cells,^7^ *SM22α* ^+^ cells.^8^ Dermal mesenchymal cells play diverse functions in skin homeostasis and wound repair by secreting extracellular matrix (ECM) molecules and soluble factors, and excessive ECM synthesis is found to inhibit hair follicle regeneration after wound healing.^9^

Dermal fibroblasts play a pivotal role in hair follicle regeneration during wound repair. Dermal fibroblast‐conditioned medium has recently been shown to induce hair regeneration,^10^ suggesting that certain fibroblasts enhances hair regeneration through a paracrine mechanism. Insulin‐like growth factor 1 (IGF1) plays a vital role in tissue homeostasis and regeneration via autocrine/paracrine/endocrine activities: it enhances skeletal regeneration and brain repair after injury in mice.^11^, ^12^ In the skin, IGF1 promotes hair follicle growth.^13^, ^14^ Several studies have shown that immune cells such as M2 macrophages and also resident epidermal T cells secrete IGF1 to enhance wound healing and hair neogenesis.^15^, ^16^ Several dermal fibroblast cell lines reportedly produce high levels of IGF1,^17^, ^18^, ^19^ but do not do not appear to correspond to known fibroblast subpopulations in vivo. Thus, while heterogeneous dermal fibroblasts are the major cell type to mediate skin regeneration after wounding, the subpopulation that produces higher levels of IGF1 has not yet been clearly determined.

We set out to identify dermal mesenchymal cells crucial for hair follicle regeneration. The surge of scRNA‐seq technology application has resulted in a large amount of publicly available scRNA‐seq data. Since neonatal skin heals with higher levels of hair follicle regeneration after wounding,^20^ we decided to start with the re‐analysis of scRNA‐seq data of neonatal mouse skin, which were reported earlier.^20^ After screening a collected database of mouse surface markers and secreting factors, we identified a mesenchymal cell subpopulation expressing higher levels of *Egfr* ^+^ and *Igf1* ^+^, which was potentially involved in hair follicle regeneration. Immunofluorescence analysis confirmed the presence of EGFR^+^ dermal fibroblasts in neonatal and adult dermis, and their co‐expression of IGF1. In addition, sorted EGFR^+^ dermal fibroblasts showed higher expression levels of *Igf1* than EGFR^−^ dermal fibroblasts. Moreover, application of IGF1 in skin wounds enhanced hair follicle regeneration in mice. Thus, our results identify that EGFR^+^ dermal fibroblasts potentially mediate skin regeneration likely through release of IGF1.

## 2 MATERIALS AND METHODS

### 2.1 Mice

*Krt14‐rtTA‐H2BGFP* mice were purchased from Jackson Laboratory. Skin GFP expression was induced by applying 0.2 mg/mL tetracycline (dissolved in 70% ethanol). Prior to topical application of tetracycline, adult mice had their dorsal hair removed by sequential application of hair clipper and hair removal cream. Twenty‐four hours later, tetracycline treated dorsal skin was ready for experiments. The animals were maintained in cages in a temperature‐controlled environment, with a 12‐h light–dark cycle and free access to food and fresh water. All animals were housed in facilities operated by the Laboratory Animal Centre of Peking University Shenzhen Graduate School. The procedures involving animals and their care were conducted following the guidelines and prior approval of the Ethics Committee of Tsinghua Shenzhen International Graduate School.

*Pdgfra Cre ERT; mTmG* mice were obtained from Dr. Ting Chen's lab. Activation of recombinase after tamoxifen injection enables the cell membrane of PDGFRα^+^ cells and its offspring to be labelled with green. 200 μL tamoxifen (Sigma, T5648) solution (dissolved in maize oil at 16.7 mg/mL) was intraperitoneally injected for five consecutive days. Mice harbouring GFP‐labelled mesenchymal cells (PDGFRα^+^) were ready for use.

### 2.2 IGF1 injection and wound model

Post Day 2 neonatal *Krt14‐rtTA‐H2BGFP* littermates were anesthetized by isoflurane inhalation (RWD Life Science Co., Shenzhen, China). Two symmetrical full thickness skin wounds were created on dorsal skin with a 2‐mm‐diameter skin biopsy punch as previously described.^39^ From post‐injury Day 7 to Day 14, each mouse received a daily injection of 50 μL PBS or murine IGF1 (MCE, HY‐P7070) solution (dissolved in PBS at 1 ng/μl) in the two wounds, respectively (beneath the scab into the wound bed tissue). Skin tissues at the wound site were collected at post‐injury Day 15 and subjected to AP staining, and skin tissues obtained at Day 21 were subjected to immunofluorescence staining.

Seven‐week‐old adult mice of the same breed and near body weight were anesthetized by intraperitoneal administration of 1% sodium pentobarbital (35 mg/kg). Mouse dorsal hair were completely removed by sequentially shaved with hair clipper, depilated with hair removal cream. Then, the dorsal skin was cleansed with wet cotton swab and sterilized with iodine solution and 75% ethanol, respectively. One 1‐cm‐diameter disinfected biopsy punch was used to create 1‐cm diameter full‐thickness wound on each mouse back. Following wounding, mice were individually housed in plastic cages. Wounds were let to heal and left with no additional dressing.

### 2.3 Dermal cell isolation

Entire dorsal skin of neonatal *Krt14‐rtTA‐H2BGFP* mice after GFP induction were collected. Pooled tissues from eight littermate mice were rinsed sequentially in an iodine solution and twice in sterile PBS. Fat tissue was scraped off with a scalpel. Sterilized skin was then incubated in 0.35% dispase II (Sigma, D4693‐1G) at 4°C overnight. The epidermis was peeled off and discarded. The remained dermis was then minced into less than 1 mm pieces, digested with 0.35% collagenase (Sigma, C0130‐1G) for 1 h at 37°C, and filtered through 70 μm and 40 μm mesh (Falcon, BD Biosciences), spun down, and resuspended in PBS. Single‐cell suspension was treated with 1x red blood cell lysis buffer, washed, and resuspended in PBS with 0.4% bovine serum albumin (BSA) (Sigma). Dead cells were removed using the MS columns of the Dead Cell Removal Kit (Miltenyi Biotec, 130–090‐101). Live cells were resuspended in PBS with 0.4% BSA and placed in 4°C for further usage.

### 2.4 scRNA‐seq data analysis

Gene‐by‐cell matrix from repeated samples was merged by Seurat v 4.0^40^ in R v4.1.2. Cells with RNA feature numbers between 300 and 8000 were passed for subsequent analysis. Run SCTransform and RunPCA function by Seurat for dimensional reduction. Cluster the skin cells with parameter (dims = 1:30, resolution = 0.2). Suspected dermal cells were extracted for recluster with parameter (dims = 1:30, resolution = 0.3). Differentially expressed genes (DEGs) generated by FindAllMarkers (logfc.threshold>0.25, min.pct = 0.1, *p* value<0.05) using the Wilcoxon rank test. DEGs of dermal cells with a high expression of the *Egfr* gene were used for GO enrichment. GO enrichment analyses were performed by R package ClusterProfiler v 3.14.^41^ To eliminate redundancy enrichment results, online tools REVIGO with default parameters was used for visualising.^42^

### 2.5 Fluorescence‐activated cell sorting (FACS)

Dermal single‐cell suspensions were prepared as described in *2.3. Dermal cell isolation*. The cells were stained for 30 minutes at 4°C with 7‐AAD Viability Staining Solution (Biolegend, 420,403) and antibodies including anti‐EGFR‐APC (Sc‐373,746, SantsCruz Biotechnology, CA, USA), anti‐CD31‐FITC (102,405, Biolegend, CA, USA), anti‐CD45‐FITC (157,214, Biolegend, CA, USA), followed by two washes in PBS mixed with 1% BSA (Sigma). Dead cells were excluded from analysis by labeling with 7‐AAD Viability Staining Solution. The FITC^+^ cells composed of unwanted epidermal cells (GFP^+^), endothelial cells (CD31^+^), and immune cells (CD45^+^) were gated out. The FITC^−^APC^+^ cells were collected as the EGFR^+^ mesenchymal cells, FITC^−^APC^−^ cells were collected as EGFR^−^ mesenchymal cells, while FITC^+^ was designated as epidermal cells. Sorting analysis was performed with BD Accuri C6 Plus and FACS Aria II (BD Biosciences). Data analysis was performed using Flowjo software (Treestar).

### 2.6 Real‐time PCR

Total RNA from FACS‐sorted cell populations from mouse skin was extracted with TRIzoL reagent (Invitrogen) quantified with the spectrophotometer (NanoDrop 2000, Thermo Scientific). Hifair III 1st Strand cDNA Synthesis SuperMix for qPCR kit (YEASEN, 11141ES60) was applied to transcribe the obtained RNA. The real‐time PCR analysis was performed with a CFX96 (Bio‐Rad) using the Hieff UNICON qPCR SYBR Green Master mix (High Rox, YEASEN 11200ES08). Every sample was tested in triplicate. Melting curves were analysed for each run to assess the presence of unspecific PCR products. Gapdh served as the housekeeping gene. The results were analysed with Bio‐Rad CFX Maestro software. The mRNA expression of tested genes was calculated relative to the expression of corresponding Gapdh based on the 2‐ΔΔ*t* method. The primers used are listed in Table S2.

### 2.7 Immunohistochemistry

Mice were sacrificed to harvest the desired skin areas. The skin was bisected along the cranial‐caudal direction, fixed in 4% paraformaldehyde (PFA) for 12 h. Fixed samples were dehydrated with 10%, 20% and 30% sucrose solution successively, embedded in optimal cutting temperature (OCT) compound and sectioned into 10‐μm pieces. The sections were washed with PBS and blocked with 3% BSA solution containing 0.1% Triton‐X 100 (Sigma) at 37°C for 1 h. The samples were incubated with primary antibodies in 1% BSA solution at appropriate concentrations (Antibodies were listed in *4.8 Antibodies*.) at 4°C for 12 h. Then the primary antibodies were removed and washed twice with PBS. The fluorescence‐conjugated secondary antibodies were applied to detect the expression of the target protein. Nuclei were counterstained with DAPI (Solarbio Life Sciences, C0060). The samples were examined under a confocal laser scanning microscope (Zeiss LSM‐780, German).

### 2.8 Antibodies

Used for immunohistochemistry, the primary antibodies included rabbit anti‐EGFR antibody (1:200 dilution, Ab52894, Abcam, Cambridge, MA, USA), rabbit anti‐IGF1antibody (1:200 dilution, Ab9572, Abcam, Cambridge, MA, USA), rabbit anti‐Vim polyclonal antibody (1:200 dilution, 10366‐1‐AP, Abcam, Proteintech Wuhan, China). Meanwhile, Alexa Fluor 488‐, 594‐, 647‐conjugated secondary antibodies were used for detection with the primary antibodies (1:200 dilution, Jackson ImmunoResearch). EGFR Monoclonal Antibody, APC (Sc‐373746, SantsCruz Biotechnology, CA, USA), CD45 Monoclonal Antibody, FITC (157214, Biolegend, CA, USA), CD31 Monoclonal Antibody, FITC (102405, Biolegend, CA, USA) were used for EGFR^+^ cells sorting according to manufacturer direction.

### 2.9 Alkaline phosphatase (AP) activity staining

According to the manufacturer's protocol, AP staining was performed using a BCIP/NBT Alkaline Phosphatase Colour Development Kit (Beyotime Biotechnology, C3206, Jiangsu, China). Frozen tissue sections were rinsed with PBS to remove OCT. The staining buffer was added to samples and incubated at 37°C for 2 h. After washing with PBS, the samples were examined and imaged under a light microscope.

### 2.10 Statistical analysis

GraphPad Prism 8.0 software was used to carry out statistical analysis of all experiments. All data were presented as means ± SEM. Paired Student's *t*‐tests were applied to compare the difference of hair follicle numbers between two groups. Unpaired Student's *t*‐tests were performed to compare expression levels between the indicated two groups. Values of *p* < 0.05 were indicative of statistically significant differences.

## 3 RESULTS

### 3.1 A distinctive EGFR ^+^ mesenchymal cell population identified in neonatal mouse skin via re‐analysis of scRNA‐seq data

**FIGURE 1:**
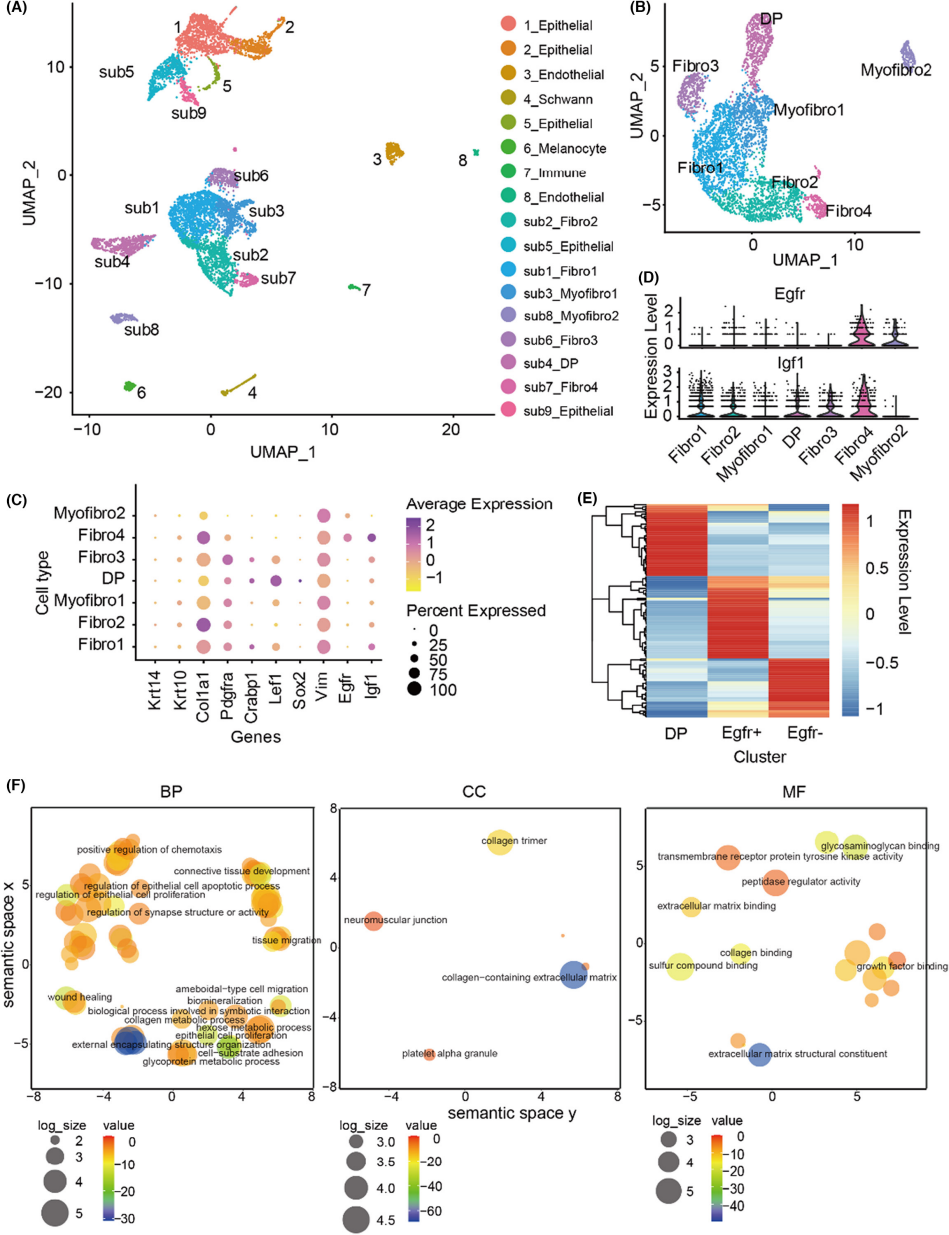
Analysis of scRNA‐seq data to identify dermal mesenchymal cell population that co‐expresses *Egfr* and *Igf1* in neonatal murine skin. (A) UMAP visualisation of all cell populations in 2‐day‐old neonatal mouse skin. Each point represents a single cell, colour‐coded based on cell types. (B) Subset and clustering of dermal cells. (C) Visualisation of dermal and epithelial marker gene expression across dermal subtypes shown in the dot plot. (D) Vlnplot showing Egfr and *Igf1* expression in dermal subclusters. (E) Heatmap of top 50 differently expressed genes in EGFR^−^ cells, EGFR^+^ cells and dermal papilla (DP) cells. (F) The GO terms of the BP, CC, and MF categories enrichment of the upregulated genes in EGFR^+^ cells. Within Cartesian coordinates (x,y), the closer the different circles are, the more related the GO terms are. The size of the circle is proportional to the number of genes within the GO terms. The colour of the circle indicates the significance enrichment. BP, biological process; CC, cellular component; GO, Gene Ontology; MF, molecular function.

To dissect the heterogeneity of fibroblasts in mouse skin, we reanalysed scRNA‐seq data from samples of P2 neonatal mouse skin.^20^ A total of 7786 sequenced cells and 18,876 genes from three libraries met the qualification threshold. We annotated with collected markers (Table S1,Figure 1A), extracted cells that expressed dermal cell marker genes and clustered them at proper resolution (Figure 1B). Fibroblast cluster 4 (Fibro4) simultaneously expressed *Egfr* and *Igf1* (Figure 1C,D), indicating that an EGFR marked subtype highly expresses *Igf1*. Dermal cells were then classified into EGFR^+^ and EGFR^−^ cells, which were transcriptionally distinct from dermal papilla (DP) cells (Figure 1E). Genes that were upregulated in the EGFR^+^ cell population were subject to gene ontology enrichment analyses; results showed that, besides gene ontology terms related to connective tissue development and epithelial cell proliferation, GO terms that include wound healing and extracellular matrix organisation were also enriched, suggesting the possible function of EGFR^+^ cells in the wound healing process (Figure 1F).

### 3.2 EGFR ^+^ dermal mesenchymal cells highly express *Igf1*

**FIGURE 2:**
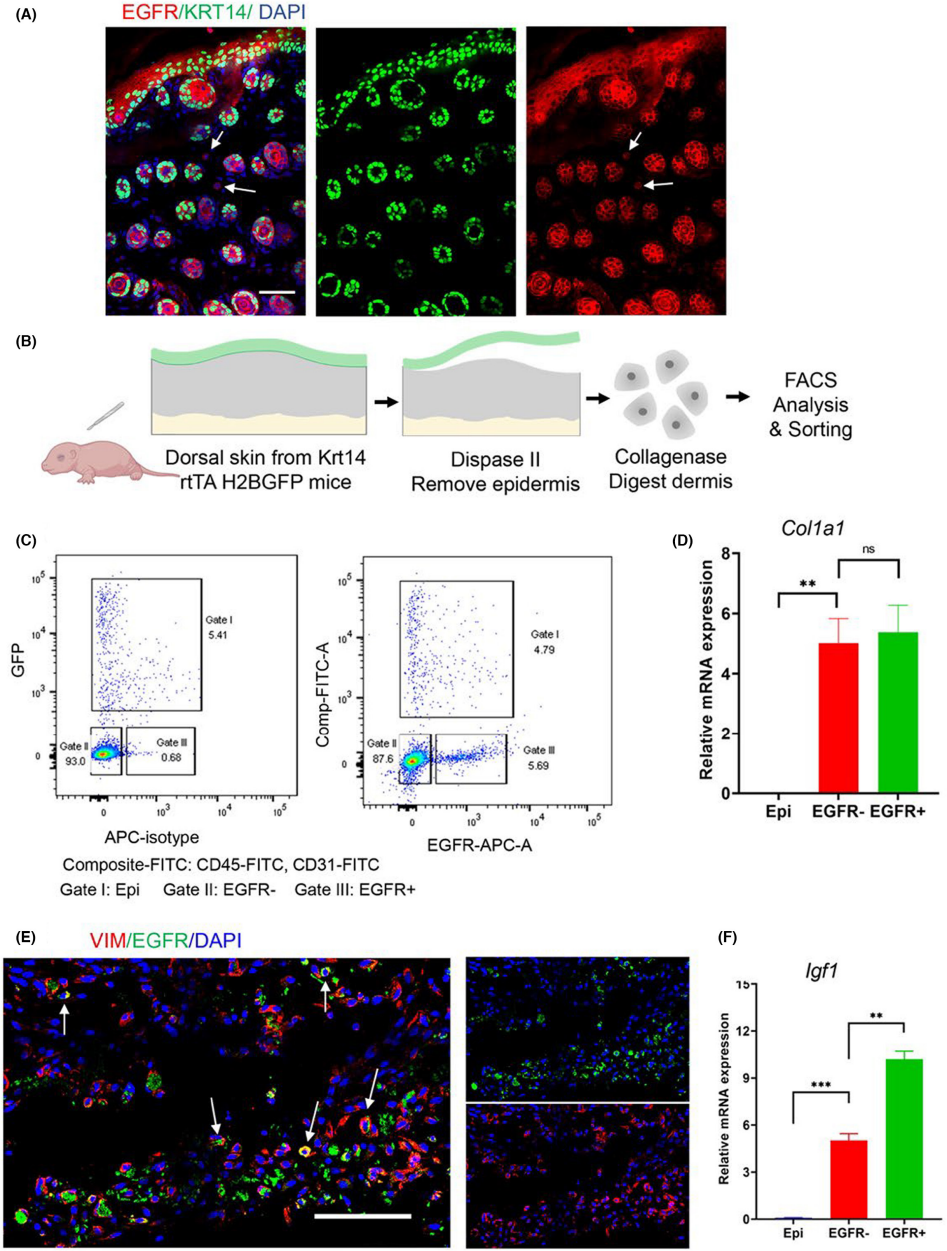
Isolation of EGFR expressing mesenchymal cells present in neonatal murine dermis. (A) Immunofluorescence staining for EGFR in tetracycline‐induced neonatal skin from Krt14‐rtTA‐H2BGFP. Scale bar: 50 μm. White arrows point to EGFR positive non‐epithelial cells. (B) Schematic illustration of our strategy for analysing and sorting of EGFR‐expressing mesenchymal cells in neonatal dermis. (C) Fluorescence‐activated cell sorting (FACS) of EGFR+ cells in compo‐site‐FITC negative cells. APC, allophycocyanin; GFP, green fluorescent protein. (D) Real‐Time PCR analysis of mesenchymal marker (Col1a1) expression in freshly isolated mouse dermal subpopulations (Data represents mean ± SEM, *n* = 3). (E) Immunofluorescence staining of EGFR, vimentin (VIM), DAPI. Scale bar, 100 μm. (F) Real‐time PCR analysis of Igf1 expression in the freshly isolated mouse dermal subpopulations. In Real‐time PCR experiments (D and F), we used triple wells for each sample. Samples from two independent experiments were examined and showed similar results. Results of one analysis are shown. **p < 0.01, ***p < 0.001; EGFR‐ and EGFR+, represent EGFR negative and positive cells; Epi, epidermal cells; ns, means no significant difference.

To establish the existence of EGFR^+^ cells in the neonatal murine dermis, we used immunofluorescence staining. As with the data analysis results, a non‐epithelial subpopulation stained positive for EGFR in neonatal murine skin (Figure 2A). To effectively exclude the EGFR‐expressing epithelial cells, we used *Krt14‐rtTA‐H2BGFP* mice that express the reverse tetracycline‐controlled transactivator (rtTA) protein under the control of the keratin 14 (KRT14) gene promoter, which provides a Tet‐On tool for KRT14 expression upon tetracycline treatment. KRT14‐expressing epidermal cells were labelled with green fluorescent protein (GFP) after being treated with tetracycline (Figure 2B). To compare the expression levels of dermal mesenchymal cells positive versus negative for EGFR, we used FACS to sort single dermal cell. A negative selection strategy to sort for EGFR^+^ mesenchymal cells was applied (Figure 2B,C), by staining a dermal cell suspension with surface marker antibodies for endothelial cells (CD31^+^) and leukocytes (CD45^+^). After excluding GFP^+^CD31^+^CD45^+^ cells (Gate I), a population with the expression of EGFR (Gate III, named EGFR^+^) and another population (Gate II, named EGFR^−^) were identified in the mouse dermis (Figure 2C). Real‐Time PCR analysis was use to show that the sorted EGFR^+^ cells with no detectable KRT14 expression (data not shown), expressed high levels of the fibroblast marker *Col1a1* in (Figure 2D). Additionally, imaging of the immunofluorescence staining showed that EGFR^+^ cells simultaneously also expressed vimentin (*Vim*) (Figure 2E), a universal mesenchymal marker. *Egfr* and *Igf1* were highly expressed in Fibro4 (Figure 1C,D). Real‐time PCR analysis of freshly isolated EGFR^−^ and EGFR^+^ cell subpopulations also showed that the expression level of *Igf1* was significantly higher in EGFR^+^ cells (Figure 2F). These results led us to conclude that EGFR marks a subpopulation of dermal mesenchymal cells with high *Igf1* expression in neonatal mouse.

### 3.3 *Igf1*‐expression colocalizes to EGFR ^+^ dermal mesenchymal cells in normal and wounded adult skin

**FIGURE 3:**
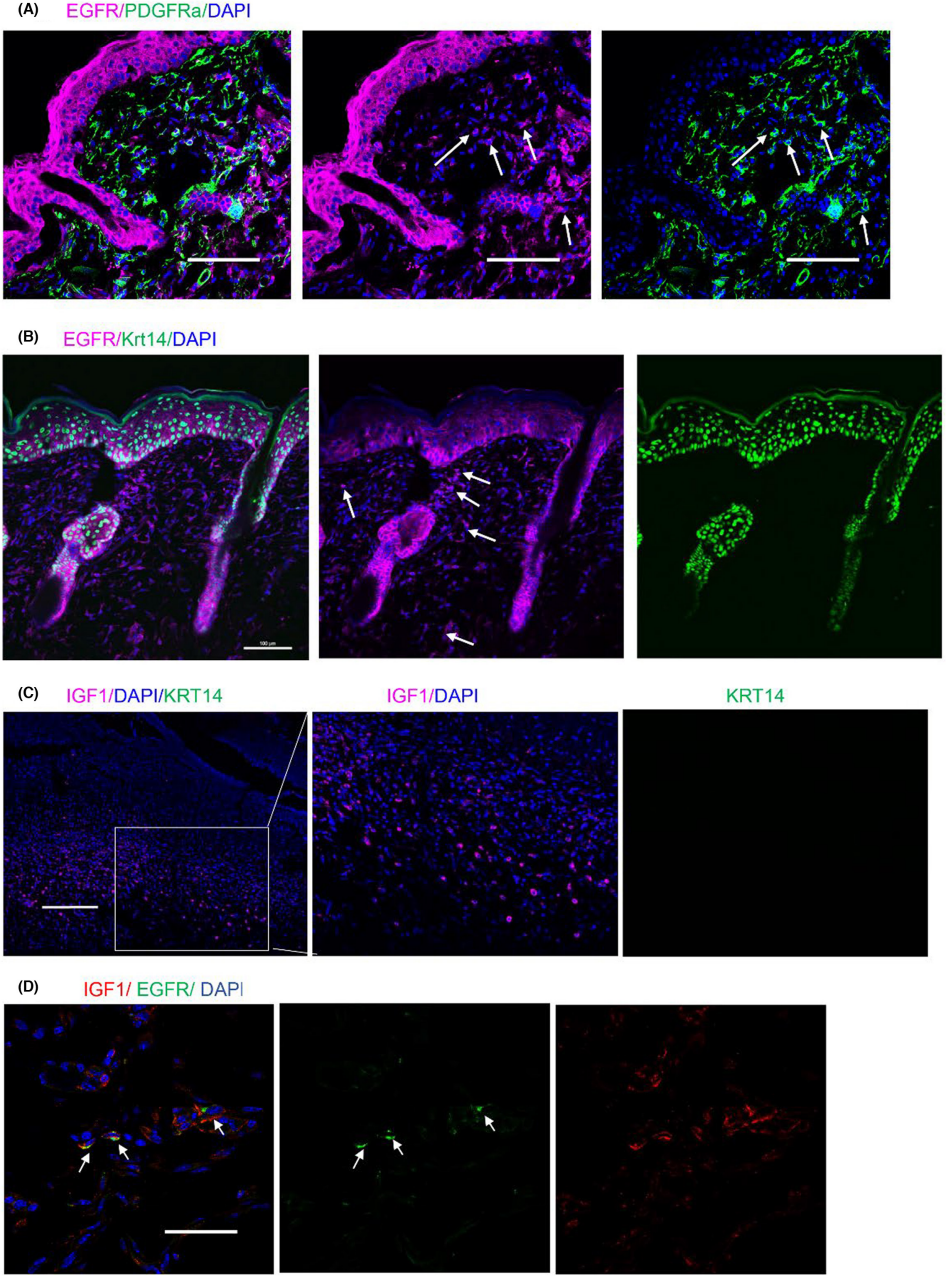
EGFR and IGF1 expression in adult murine dermis. (A) Immunofluorescence staining for EGFR and DAPI in skin tissues of 7‐week‐old *PDGFRa cre ERT;mTmG* mice . Scale bar, 100 μm. (B) Immunofluorescence staining of EGFR in tetracycline‐induced *Krt14‐rtTA‐H2BGFP* adult mouse skin. White arrows point to EGFR positive non‐epithelial cells. Scale bars, 100 μm (C) Immunofluorescence staining of IGF1 in Day 7 wounds. Scale bars, 200 μm. (D) Co‐localisation of IGF1 and EGFR in adult wound dermis. White arrows point to EGFR positive cells. Scale bars, 100 μm.

We next used immunofluorescence staining to detect the expression of EGFR in adult mouse dorsal skin. In adult *pdgfra‐GFP* mice, dermal fibroblasts are labelled by GFP. Immunofluorescence staining for EGFR detected cells double positive for GFP and EGFR in the dermis (Figure 3A). In *Krt14‐rtTA‐H2BGFP* adult mice, partial non‐epithelial cells were positively stained with EGFR which distributed across the dermis (Figure 3B). It was reported that during wound healing, IGF1 signalling mainly functions during the granulation and epithelialisation stage.^21^ We stained IGF1 in adult wounds. IGF1^+^ cells were mainly distributed in the lower layer of the wound bed in Day 7 wounds (Figure 3C), and some of them were colocalized to EGFR^+^ cells (Figure 3D). The results indicated that the EGFR^+^ mesenchymal cells participated in wound healing by highly expressing *Igf1*.

### 3.4 IGF1 enhances wound‐induced hair neogenesis

**FIGURE 4:**
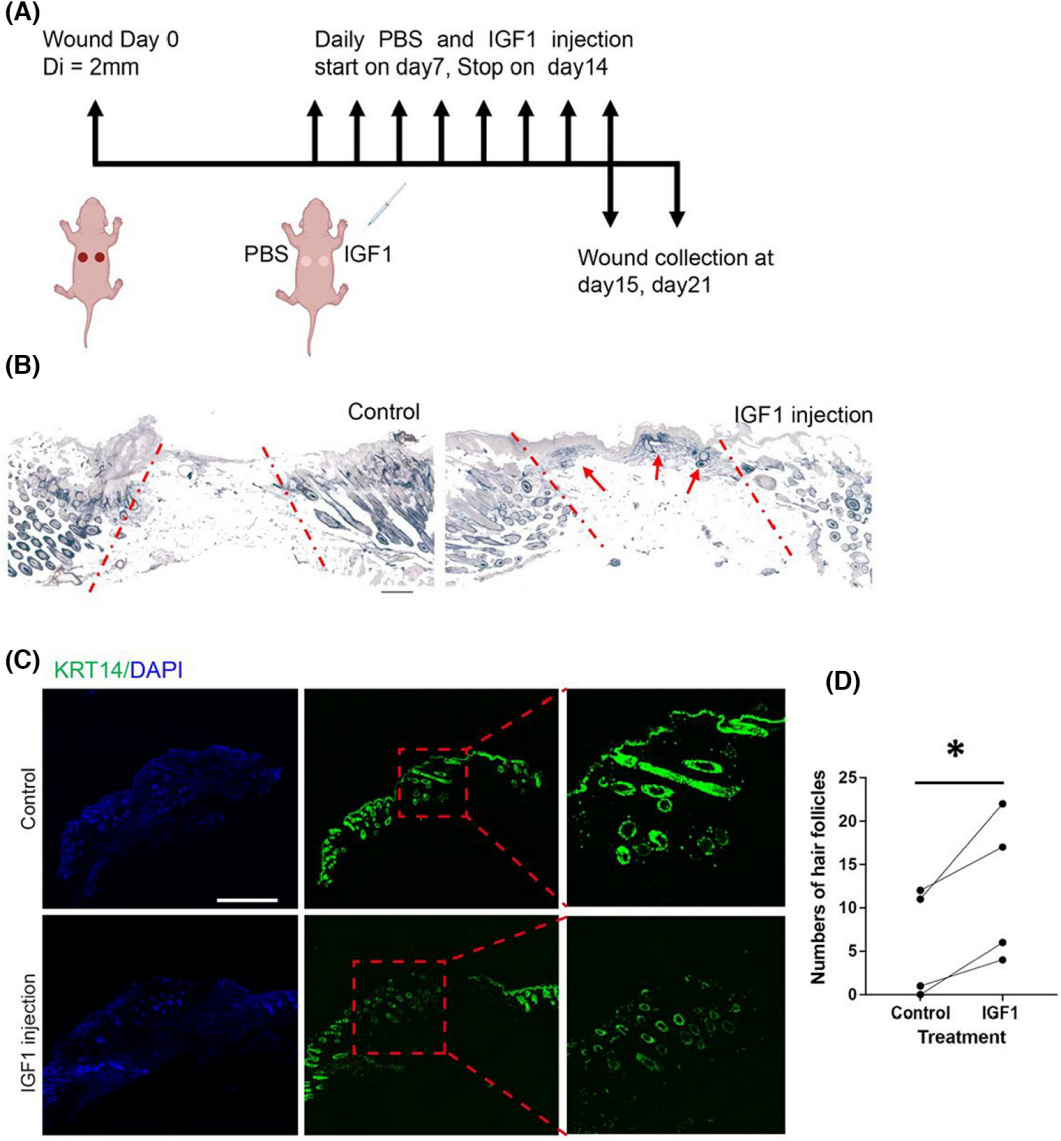
Effects of IGF1 on wound‐induced hair follicle neogenesis. (A) Schematic representation of strategy for exogenous IGF1 injection during wound healing process. Two full thickness wound (diameter = 2 mm) were made on neonatal mouse dorsal skin. From post‐wounding Day 7 to Day 14, each mouse received a daily injection of 50 μL PBS or murine IGF1 solution (dissolved in PBS at 1 ng/μl) beneath the scab into the wound bed tissue in the two dorsal wounds, respectively. (B) AP staining on wound tissue from control group (PBS) and IGF1 injection group collected at post wound Day 15. The wound bed lies between red dash lines in each picture. AP was stained blue. Red arrows point to AP positive area including neogenic hair follicles. Scale bar, 200 μm. (C) Visualisation of hair follicles in healed wounds collected at Day 21. (D) Statistics of hair follicles in both groups collected at Day 21. Data represents the mean ± SEM, *n* = 4, *p < 0.05.

Next, exogenous IGF1 was tested if it facilitates wound‐induced hair neogenesis (WIHN). In vivo application of exogenous IGF1 was conducted by daily injecting 50 μL mouse IGF1 solution (1 ng/μL) or PBS into the centre of the wound (beneath the scab) from post wound Day 7 to Day 14, as illustrated in Figure 4A. PBS or IGF1 treated wound tissues, collected on post wound Day 15 and Day 21, were subjected to the frozen section. The results of alkaline phosphatase (AP) staining on post wound Day 15 tissues indicated that the area size of AP positive in the IGF1 injection group was larger than in the PBS treated group (Figure 4B). Several hair follicles were formed in the IGF1 treated group (Figure 4B). Considering that the neogenic hair follicles in healed wounds have irregular directions, it was barely possible to have every hair follicle in complete shape in one frozen section slice. Each circular structure formed by the KRT14^+^ cells in the wound bed was considered as one hair follicle (Figure 4C). Paired Student t‐test statistics was employed to investigate the discrepancy of hair follicle numbers between two groups (Figure 4D). The number of hair follicles that grew in the IGF1 treated group was significantly larger than the control group at post wound Day 21 (Figure 4C,D). The results indicate that exogenous IGF1 promoted WIHN.

## 4 DISCUSSION

Despite the presence of diverse subpopulations of fibroblasts in the dermis, the functional heterogeneity of these cells remains largely unknown. A number of studies have used scRNA‐seq to identify subpopulations in skin from humans and mice.^3^, ^22^ We tried to take advantage of the amounted scRNA‐seq data to identify dermal mesenchymal population that is important for hair follicle regeneration. Together with experimental studies, we identified that EGFR marks a subpopulation of dermal mesenchymal cells highly expressing IGF1 which enhances hair follicle regeneration.

Mesenchymal cells play a variety of roles primarily by secreting ECM and soluble substances. As soluble factors such as growth factors and cytokines are critical components of stem cell niches, here we have focused on identifying mesenchymal stem cells that express high levels of secreted factors. Using a two‐marker screening approach, we have documented that cell surface protein EGFR marks a mesenchymal subpopulation that highly expressed *Igf1* in the dermis of neonatal and adult mice. Human dermal fibroblasts reportedly stain positive for EGFR.^23^ Our results display that EGFR is also distributed across the murine dermis (Figures 2A and 3A), similar to its distribution in human skin (The Human Protein Atlas database). In addition, EGFR is expressed in the human fibroblast cell line Hs68, and the level of EGFR expression decreases with cell aging,^24^ which might lead to its reduced responsiveness to epidermal growth factor (EGF). Furthermore, human dermal fibroblasts produce IGF1,^25^, ^26^ yet but their molecular identity remained unknown. We have demonstrated that EGFR^+^ dermal fibroblasts express higher levels of IGF1 than EGFR^−^ fibroblasts.

Our findings point to the possible roles of EGFR^+^ cells in the human dermis. To obtain EGFR^+^ mesenchymal cells, we employed a negative selection strategy, as per earlier studies,^27^ in which GFP and FITC^+^ cells, including GFP^+^ epidermal cells, CD45^+^ immune cells and CD31^+^ endothelial cells were removed (Figure 2B). It seemed that the strength of EGFR^+^ signal arrows pointed to was much weaker than that of the surrounding epidermal part (Figure 2A), which could be the result of the fact that signal strength from a single mesenchymal cell did not match that of concentrically aligned epidermal cells. It could also be EGFR expression in the mesenchymal cell was significantly lower than that in the epithelial cells. We learned from the dot plot (showing in Figure 1C) that, in addition to Fibro4, the designated EGFR^+^ cell population, other populations with high *Igf1* expression but low EGFR expression Fibro3, Fibro1, and Fibro2 were also present. However, *Igf1* mRNA levels were much higher in EGFR^+^ mesenchymal cells than in the control group. These findings suggested that dermal cells expressing *Igf1* are primarily composed of EGFR^+^ mesenchymal cells.

IGF1 is a dermis‐derived growth factor that regulates growth, survival, and differentiation in many tissues via IGF1/IGF1R signalling. Dermal fibroblast cell lines secrete high levels of IGF1,^17^, ^18^, ^19^ yet the corresponding in vivo subpopulations remain unknown. IGF1 plays an important role in the growth of hair follicle, both under homeostasis and after disruption of skin integrity. Local application of exogenous IGF1 increases the number of hair follicles and prolongs their growth phase.^14^ Forced expression of *Igf1* in mouse keratinocytes accelerates hair follicle formation and cycling.^28^ High level of IGF1 protein in the early phase of wound healing in pigs,^29^ humans^30^ and rats^31^, ^32^ which could probably derive from blood,^33^, ^34^ resident epidermal T cells,^15^ M2 macrophages^16^ and fibroblasts. Despite there being circulating IGF1 in the blood, local IGF1 is more likely to contribute to a niche specific for hair follicle neogenesis. Several dermal fibroblast cell lines produce IGF1, but they do not correspond to known fibroblast subpopulations in vivo. Our study revealed that a subpopulation of dermal fibroblasts expresses higher levels of IGF1, that are positive for EGFR, suggesting the potential role of fibroblasts secreted IGF1 in hair follicle regeneration. Wounds treated with IGF1 exhibit a larger size of the AP‐positive area (Figure 4B) along with larger numbers of hair follicles that have regenerated in the wound (Figure 4C,D). This is consistent with earlier studies on the roles of IGF1 in promoting WIHN.^16^ Also, besides being present in epidermal cells, IGF1‐R is found in fibroblast‐like cells that are dispersed throughout the uppermost regions of the dermis,^26^ where the fibroblasts are believed to play a supportive role in hair follicle regeneration.^1^ In addition, the IGF1‐R‐related signal is involved in protecting cells from apoptosis during wound healing,^35^ potentially contributing to hair follicle regeneration. In Figure 3C, the number of IGF1^+^ cells is greater than the number of EGFR^+^ cells, suggesting that besides EGFR^+^ mesenchymal cells, other cells such as M2 macrophage^16^ that are located in wounds also likely secrete IGF1.

Our study has several limitations. (i) The characterisation of EGFR^+^ mesenchymal cells both in vitro and in vivo can be improved. However, after we removed CD45^+^ immune cells and CD31^+^ endothelial cells, notably high expression of Col1a1, a mesenchymal marker, and the co‐localisation of EGFR and VIM provided sufficient evidence to identify the EGFR^+^ cells as mesenchymal cells. (ii) Experiments on loss‐of‐function analysis of EGFR^+^ dermal fibroblasts support their role in hair follicle regeneration. Mouse skin in which epidermal EGFR was depleted displayed lesions similar to skin rashes seen in human,^36^ while the effect of ablating dermal EGFR has not yet been evaluated. Because systemic knockout of *Egfr* is fatal in mice,^37^ future studies in mice can selectively induce the deletion of the gene in fibroblasts and observe their impact on hair follicle regeneration. (iii) The role of IGF1 derived from dermal fibroblasts can be further determined by additional experiments. For example, EGFR^+^ dermal cells can be isolated and their effect on hair follicle regeneration evaluated with use of a hair follicle reconstitution assay. However, the yield of sorting EGFR^+^ dermal cells is very low, so a large number of mice would be needed to obtain a sufficient number of the EGFR^+^ dermal cells. In addition, we used recombinant IGF1 to induce hair follicle regeneration, since the bioactivity of recombinant IGF1 is similar to that of endogenous IGF1^38^; however, recombinant IGF1 may not fully simulate the function of IGF1 that is released by dermal fibroblasts.

Our finding of the dermal EGFR^+^ mesenchymal cell subpopulation that highly expresses IGF1 and enhances cutaneous regeneration has increased the current understanding to mesenchymal cell biology. Mesenchymal cells exist widely in many tissues in our body. Therefore, in future studies it is of interest to investigate whether some of them also express IGF1 and have an enhanced role in tissue haemostasis and regeneration. In addition, our study has suggested a therapeutic potential of the EGFR^+^ mesenchymal cells in wound repair. On the other hand, it is also of significance to examine whether a similar population of mesenchymal cells exists in tumours and their activities in tumorigenesis and progression.

In summary, we have used a double marker screening approach in combination with animal experiments to identify an EGFR^+^ mesenchymal population that expresses high levels of *Igf1* in neonatal and adult mouse dermis, which are potentially involved in hair follicle regeneration.

## AUTHOR CONTRIBUTIONS

**Min Chen:** Conceptualization (lead); data curation (lead); investigation (equal); writing – original draft (lead). **Zaoxu Xu:** Formal analysis (lead); software (lead). **Yu Chen:** Conceptualization (equal); validation (equal). **Qingyang Yang:** Data curation (equal); methodology (equal). **Ruiqing Lu:** Data curation (equal); validation (equal). **Yankai Dong:** Conceptualization (equal); validation (equal). **Xiaosong Li:** Conceptualization (equal); investigation (equal). **Jundong Xie:** Data curation (equal); investigation (equal). **Ren‐He Xu:** Methodology (equal); supervision (equal). **Haidong Jia:** Resources (equal). **Yan Kang:** Resources (equal). **Yaojiong Wu:** Conceptualization (lead); project administration (equal); supervision (equal); visualization (equal); writing – review and editing (lead).

## FUNDING INFORMATION

This work was supported by grants from the Natural Science Foundation of China (31961160702), Shenzhen Science and Technology Innovation Committee (WDZC20200820173710001, JCYJ20190809180217220, KCXFZ20201221173207022), State Key Laboratory of Chemical Oncogenomics fund, and Tsinghua Shenzhen International Graduate School Overseas Cooperation Fund (HW2021003).

## CONFLICT OF INTEREST STATEMENT

The authors indicate no potential conflicts of interest.

## Supporting information


Appendix S1

## Data Availability

The data underlying this article will be shared on reasonable request to the corresponding author.
