# Supplementary material for: EGFR marks a subpopulation of dermal mesenchymal cells highly expressing IGF1 which enhances hair follicle regeneration
Source: J Cell Mol Med. 2023 May 10;27(12):1697–707. doi: 10.1111/jcmm.17766 (PMC10273066; doi:10.1111/jcmm.17766)
Supplement: Supplementary file 1 — Appendix S1 [file JCMM-27-1697-s001.docx]

**Supplementary Materials:**

| **Cell types** | **Markers** |
| --- | --- |
| Dermal fibroblasts | *Col1a1, Pdgfra, Col3a1,* |
| Epidermal cells | *Krt14, Krt15, Krt10, Lgr5* |
| Immune cells | *Ptprc, Lyz2, Cd52* |
| Endothelial cells | *Pecam1, Lyve1, Kdr* |
| Melanocyte | *Tyr, Tyrp1, Dct* |
| Schwann cells | *Cadm4, Gpm6b, Plp1* |
| Myofibroblast | *Cald1, Myl9, Tagln, Acta2* |

**Table S1.** Markers used for cell population characterization

**Table S2.** Primers for Real-Time PCR

| **Gene** | **Forward primer** | **Reverse primer** |
| --- | --- | --- |
| Gapdh | AGGTCGGTGTGAACGGATTTG | TGTAGACCATGTAGTTGAGGTCA |
| Col1a1 | CCTCAGGGTATTGCTGGACAAC | CAGAAGGACCTTGTTTGCCAGG |
| Igf1 | GTGGATGCTCTTCAGTTCGTGTG | TCCAGTCTCCTCAGATCACAGC |
